# Primary urachal adenocarcinoma treated with partial cystectomy - Case report

**DOI:** 10.1016/j.ijscr.2025.111449

**Published:** 2025-05-16

**Authors:** Chale Yohannes Tegegne, Fitsum Solomon Bekele, Abiy Tadele Alene, Kinfe Tsehaye Gebreegziabher, Amanuel Damie Jiffar, Suleman Essa Ahmed

**Affiliations:** Addis Ababa University, School of Medicine, Addis Ababa, ET, Ethiopia

**Keywords:** Urachus, Urachal adenocarcinoma, Partial cystectomy, Chemotherapy

## Abstract

**Introduction:**

Urachal adenocarcinoma is a rare non urothelial malignancy which arises from the urachus, which is a fibrous remnant of allantois that extends from the bladder to the umbilicus.

**Case presentation:**

A 36 years old man presented with haematuria of 3 months duration. Cystoscopy revealed single solid growth at the dome of the bladder. Transurethral resection (TUR) biopsy showed invasive adenocarcinoma, on pelvic MRI there was 5 cm ∗ 3 cm bladder dome mass. Partial cystectomy with en-bloc resection of the urachus and umbilicus was done. The pathology result becomes mucinous adenocarcinoma and surgical margins were free.

**Discussion:**

Patients usually present with nonspecific symptoms. The most common manifestation is haematuria. Due to late symptomatic presentation it has poor prognosis. The mainstay of management is partial or radical cystectomy with en-bloc resection of the urachus and umbilicus. The role of adjuvant chemotherapy needs to be established.

**Conclusion:**

Primary urachal adeno carcinoma is a rare disease. Due to the rarity of the case there is no standard clinical guideline for the management of urachal cancer. Its tendency for local invasion, late symptomatic presentation, and metastasis can lead to a poor prognosis. Timely diagnosis and early partial or radical cystectomy with enbloc resection of the urachus and umbilicus is critical for the survival of the patient.

## Highlights

• PUA is a rare non urothelial tumour which arises from the urachus.
• Majority of cases are asymptomatic and present with advanced stage.
• Partial cystectomy with en-bloc resection of the urachus and umbilicus is the mainstay of management.
• The importance of chemoradiotherapy is not yet well established.
• Recurrence is common in PUA and close followup is mandatory.

## 1 Introduction

The urachus is a 5 cm to 10 cm long vestigial band of musculofibrous tissue that forms around week four of embryonic development. It connects the allantois to the early fetal bladder. The canal typically seals off around month four and became median umbilical ligament [1]. But can remain patent in small proportion of adults [2,3]. This is typically asymptomatic and usually only detected incidentally, and if symptomatic, it can present with congenital anomalies with leakage of urine from the umbilicus [3]. However, there is no known association between maintenance of this communication and the ultimate development of urachal cancer [2].

Primary urachal adenocarcinomas (PUA) is rare, occurring in only 0.01 % of all adult cancers and <1 % of all bladder carcinomas [2,3]. It is an aggressive malignant tumor affecting mostly men in the V–VI decade of age [4]. Its tendency for local invasion, late symptomatic presentation, and metastasis can lead to a poor prognosis. Timely diagnosis is crucial, and surgical intervention remains the cornerstone for treatment [3].

## 2 Case presentation

A 36 years old police officer from Addis Ababa came to our urology outpatient department after presented with hematuria of 3 months duration associated to this he had urinary frequency. Otherwise no abdominal swelling or discharge from the umbilicus. No history of bowel complaint. No history of smoking. The patient had no other medical comorbidities.

On physical examination vital signs were in the normal range. No palpable abdominal mass, no discharge from the umbilicus. No lymphadenopathy.

On investigation, haemoglobin was 12.7 g/dl, liver function test and renal function tests were normal. Urine dipstick was positive for blood (+3).

**Fig. 1:**
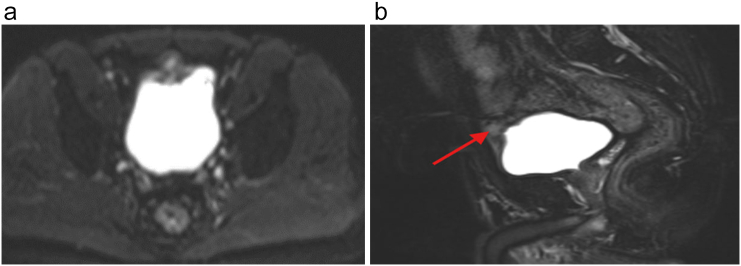
MRI of the patient shows bladder dome mass.

Cystoscopy showed single solid growth at the dome of the bladder. Transurethral resection of bladder tumor (TURBT) was done and biopsy showed invasive adenocarcinoma. Pelvic MRI showed 5 cm ∗ 3 cm mass at the dome of the bladder (Fig. 1A and B).

Case was discussed with the patient including the management options and agreed for partial cystectomy.

**Fig. 2:**
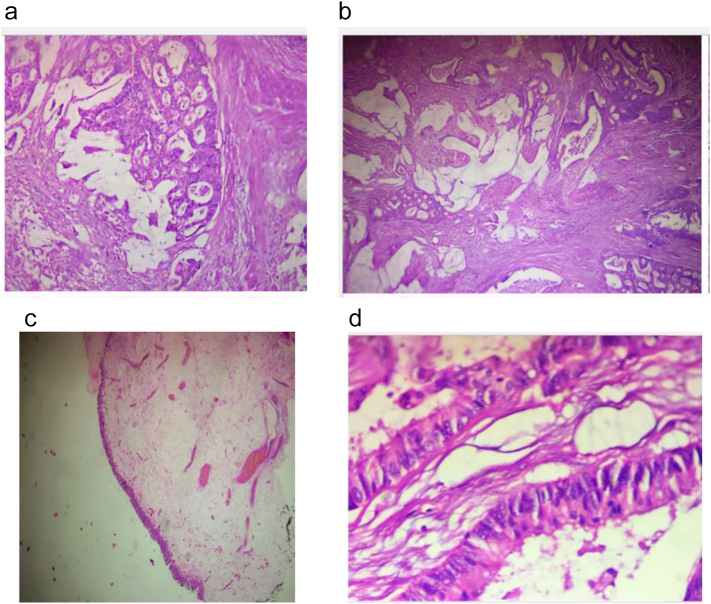
H & E sections show variable sized malignantglands lined by pleomorphic columnar cells with associated mucin production, infiltrating the muscle proper of the bladder wall. The overlying bladder epithelium appears normal with no intestinal metaplasia.

Circumferential incision made around the umbilicus and incision extended vertically midline to the level of the pubis. There was 4 cm ∗ 5 cm solid mass at the dome of the bladder which was continuous with the urachus. Circumferential incision made on the bladder 2 cm away from the tumor. The tumor with urachus and umbilicus excised en-bloc. Bladder closed in layers. There was no enlarged lymph node. Biopsy turned out to be mucinous adenocarcinoma PT2 (Fig. 2a, b, c, d), and surgical margins were free. Cystoscopy done at three months post-surgery was normal finding and the bladder capacity was 350 ml. On subsequent follow-up patient had no symptoms. He had oncology follow-up and opted for active surveillance.

## 3 Discussion

PUA is a rare malignancy occurring in only <1 % of all bladder carcinomas [1,2]. There is no known risk factor for the occurrence of urachal cancer. There are two theories regarding the development of urachal tumors. The first one is it may arise from enteric rests which are left behind in the cloaca during embryologic development. An alternative proposal is that it may arise from a metaplastic pathway [2].

Since the urachal ligament lies outside the bladder, most patients present with locally advanced disease. In early stages, this cancer is typically asymptomatic. Patients experience hematuria only once sufficient burden of tumor has developed and eroded through the bladder wall [2]. Less frequently, patients can present with abdominal pain, dysuria, mucosuria, nonspecific urinary tract disturbance (pyuria, pollakiuria, chronic urinary tract infections), discharge from the umbilicus, or with systemic nonspecific symptoms (fever, weight loss, nausea) and palpable supra pubic mass [4]. Urachal carcinoma is associated with a poor prognosis that is considerably worse than that of primary bladder adenocarcinoma [5]. The tumor has a predilection for local invasion, most often to the space of Retzius, peritoneum, abdominal wall and bladder [5].

Its tendency for local invasion, late symptomatic presentation, and metastasis can lead to a poor prognosis, with mean survival of 12–24 months for locally advanced or metastatic disease [3].

Diagnostic modalities include cystoscopy which localizes the tumor site and transurethral resection of the tumor confirms an adenocarcinoma in the midline of the bladder. Ultrasound, computed tomography (CT) and magnetic resonance imaging (MRI) are the diagnostic imaging modalities. The presence of a midline mass in the bladder that is solid or cystic, especially with small calcifications, is considered a pathognomonic finding [2]. Radiographic imaging is also helpful in determining the extent of disease and whether the tumor is metastatic or potentially surgically resectable [2].

According to Davide L et al., diagnostic criteria have been suggested to standardize the urachal cancer diagnosis. These include the following.

1. Tumor located in the dome or anterior bladder wall.
2. Tumor growth in the bladder wall.
3. Absence of atypical intestinal metaplasia and cystitis/glandularis beyond the dome/anterior wall.
4. Absence of a urothelial bladder neoplasia.
5. Exclusion of a primary adenocarcinoma of a different origin.

Presentation of an adenocarcinoma of the bladder occurring anywhere along the midline in addition to the bladder dome should be considered an urachal tumor unless proven otherwise [2].

Majority of urachal cancers are invasive adenocarcinoma [1,2,4]. Among the histology subtypes, the most frequently reported is the mucinous adenocarcinoma followed by intestinal, and mixed types [4]. Our case was mucinous adenocarcinoma.

There are different staging systems including Mayo staging, Sheldon staging system and TNM staging systems [4]. According to Sheldon staging system our case is stage IIIA since there is involvement of the bladder wall with no lymph node or distal metastasis.

Because of its rare occurrence, there have been no large, randomized, prospective trials evaluating urachal cancers, and therefore there are limited evidence-based guidelines on disease management.

The mainstay of management of urachal adenocarcinoma is partial cystectomy or radical cystectomy with en bloc resection of the urachus and umbilicus [1,6]. Bilateral pelvic lymph node dissection (PLD) can be done for staging but has no overall survival benefit [7].

There is yet no proven role for neoadjuvant or adjuvant chemotherapy, though combinations of 5-fluoruracil with cisplatin are active in those with metastases [2]. Urachal cancers are not radiosensitive, and therefore radiotherapy is seldom utilized [3]. After surgery, long-term survival is associated with negative surgical margins and absence of nodal involvement [4]. In our case we did .partial cystectomy with en-bloc resection of the urachus and the umbilicus, and the margins are free, and there was no lymphadenopathy. The patient is on active surveillance.

Urachal adenocarcinoma has a high recurrence rate. Most common sites of recurrence are the pelvis, bladder, lungs, and wound site or abdominal wall [3,4]. Lack of en-bloc resection of the umbilicus with the urachal ligament is one of several risk factors increasing the risk of relapse following surgery [2]. In many cases, local recurrence occurs within 2 years after surgery [8].

More than 20 % of urachal carcinomas have distal metastasis at initial presentation and about 59 % of cases will have distal metastasis at some point in time of the evolution of the disease [3]. Lung, bone, peritoneum, liver, iliac and inguinal lymph nodes are the most involved organs [3,7]. This case report is written based on SCARE guideline [9].

We revised different case reports about PUA and we put a summarized table below (Table 1).

**Table 1:** Review of literatures (case reports) on PUA.

Reference	Age	Sex	Main clinical presentation	Surgical management	Chemotherapy/Radiotherapy
[10]	47	M	Hematuria	Partial cystectomy	No
[11]	49	F	Abdominopelvic mass	Partial cystectomy + hysterectomy + bilateral salpingectomy + ileac lymph node biopsy	No
[8]	41	F	Abdominal mass	Partial cystectomy	Took adjuvant chemotherapy
[3]	40	M	Hematuria	Partial cystectomy + extended pelvic lymphadenectomy	Took 8 cycles of chemotherapy(FOLFOX)
[12]	60	M	Weight loss	Partial cystectomy	No
[13]	40	F	Recurrent urinary tract infections	Partial cystectomy	Took chemotherapy after 9 months due to metastasis (peritoneal carcinomatosis)
[14]	56	M	Dysuria	Partial cystectomy	Took adjuvalt chemotherapy and radiotherapy
[15]	50	F	Fungating abdominal mass	Partial cystectomy with en-bloc excision of abdominal wall	No
[16]	48	M	Hematuria	Partial cystectomy	No
[17]	15	M	Hematuria	Partial cystectomy	No
[18]	34	M	Abdominal pain	Not mentioned	Not mentioned
[19]	13	F	Abdominal pain	Partial cystectomy + segmental ileal resection + right salpingo-opherectomy	Took 4 cycles of adjuvant chemotherapy (gemcitabine and cisplatin)
[20]	58	F	Hematuria	radical cystectomy with incontinent ileal conduit, along with hysterectomy, bilateral salpingo-oophorectomy, anterior vaginal wall excision, and appendectomy	No
[21]	67	–	No symptom(incidental finding)	Urachus treated with umbilical-sparing robotic partial cystectomy.	No
[22]	52	M	Shortness of breath and weight loss	Surgery was not done Since it was advanced, metastatic to bone marrow, lung and liver	FOLFOX-6
[23]	40	M	Hematuria and abdominal pain	Partial cystectomy	No
[24]	73	F	Hematuria	Partial cystectomy with pelvic lymph node dissection	No
[25]	46	M	-Hematuria	-Not done due to lung metastasis	-FOLFOX 3 cycles.
	50		-Hematuria	-not done due bone and lymph node metastasis	-Capecitabine

## 4 Conclusion

Urachal cancer is a rare and aggressive malignant tumor for which limited evidence is available to guide clinicians in diagnosis and treatment. Early diagnosis with complete en bloc resection of the tumor with urachus and umbilicus is the mainstay of management. Role of adjuvant and neo adjuvant chemotherapy is not yet established. Considering the rarity of the case, it is worth reporting new case of primary urachal adenocarcinoma.

## Author contribution

1: Chale Yohannes Tegegne (urology resident): Conceived, wrote the original draft, edited and submitted the report.
2: Fitsum Solomon Bekele: Operated on the patient, edited the report.
3: Abiy Tadele Alene (urology resident): Operated on the patient and participated on writing the draft of the report.
4: Kinfe Tsehaye Gebreegziabher (urology resident): Participated on the operation, followed the patient, edited the report.
5: Amanuel Damie Jiffar (pathologist): Evaluated the specimen.
6: Suleman Essa Ahmed (pathology resident): Evaluated the specimen, contributed the histologic images.

## Consent

Written informed consent was obtained from the patient for publication of this case report and accompanying images. A copy of the written consent is available for review by the Editor-in-Chief of this journal on request.

## Ethical approval

Ethical approval was provided by the author's institution.

Ethical review committee of the Department of Surgery, College of Health Sciences, School of Medicine, Addis Ababa University, Addis Ababa, Ethiopia on February 5, 2025.

## Guarantor

Chale Yohannes Tegegne (urology resident).

## Research registration number

N/A.

## Funding

This case report has no any source of funding.

## Clinical trial number

Not available.

## Conflict of interest statement

Authors declared that there is no any conflict of interest.

## Data Availability

The data set used and/or analysed during this case report are available from the corresponding author on request.
